# Multicomponent prehabilitation and perioperative care in older persons with frailty referred to planned orthopaedic surgery: A feasibility study

**DOI:** 10.1007/s40520-025-03214-1

**Published:** 2025-11-06

**Authors:** Camilla Blach Rossen, Anne Mette Schmidt, Maria Stokholm Hansen, Anne-Louise Degn Wivelsted, Peter Vedsted, Merete Gregersen

**Affiliations:** 1 Elective Surgery Center Silkeborg Regional Hospital, University Clinic for Interdisciplinary Orthopaedic Pathways (UCOP), Regional Hospital Central Jutland, Silkeborg, Denmark; 2 Department of Clinical Medicine, Aarhus University, Aarhus, Denmark; 3 Medical Diagnostic Centre, University Clinic for Innovative Patient Pathways, Regional Hospital Central Jutland, Silkeborg, Denmark; 4 Department of Geriatrics, Aarhus University Hospital, Aarhus, Denmark

**Keywords:** Denmark, Geriatric assessment, Geriatric nursing, Perioperative care, Preoperative exercise, Orthopaedics, Feasibility studies.

## Abstract

**Purpose:**

To assess the feasibility and acceptability of a multicomponent prehabilitation and perioperative care intervention for older persons with frailty scheduled for elective orthopaedic surgery prior to conducting an RCT.

**Methods:**

A mixed-methods feasibility study assessed *reach*,* dose*,* data collection procedure*,* acceptability* and *adaptation*. Data was collected through patient reported outcomes, electronic medical record, and interviews with patients and healthcare professionals. No pre-defined feasibility criteria were applied.

**Results:**

Of those referred for surgery, 30% were screened for frailty, among those eligible, 78% consented to participate. Participants received six of the eight planned phone calls, adherence to nutrition advice and exercise was high, and medication review resulted in at least one drug change for 50% of participants. Qualitative analysis identified key themes including the importance of tailoring care to individual patient needs, strong support from healthcare professionals and family members, and effective interdisciplinary collaboration. The intervention appeared feasible and acceptable to both patients and healthcare professionals. Several adaptations were implemented immediately (refined recruitment procedures, reduced number of health coaching sessions, and modified data collection method), while others were proposed (earlier involvement of geriatrician, focusing on hip and knee surgery, 48-hour follow-up, and improved integration with municipal rehabilitation services and general practitioners).

**Conclusion:**

This study demonstrated the feasibility and acceptability of a prehabilitation and perioperative care intervention for older persons with frailty undergoing elective orthopaedic surgery. The proposed adaptations will inform the implementation strategy prior to conducting an RCT to evaluate effects on clinical outcomes and healthcare costs.

**Key summary points**.

Aim: To evaluate the feasibility and acceptability of a multicomponent prehabilitation and perioperative care intervention for older persons with frailty undergoing elective orthopaedic surgery.

Findings: The intervention was feasible and well-received, with individualised support and interdisciplinary collaboration. Adaptations were identified to support future implementation.

Message: The adjusted intervention for prehabilitation of older persons with frailty in surgical preparation and recovery is now ready for implementation, followed by an RCT.

## Introduction

Older persons face an enhanced risk of accelerated disease progression and functional decline during surgery. Frailty further compounds this risk, with older persons with frailty spending more days in the hospital after undergoing surgery and experiencing a higher likelihood of complications compared to their non-frail counterparts [1–3]. Frailty, a multidimensional syndrome, is characterised by decreased physiological reserves, making patients susceptible to stressors [4, 5]. However, some factors contributing to the negative effects of frailty are modifiable and can be addressed [6]. Aiming to provide person-centred care [7], identifying frailty prior to surgery, and tailoring a perioperative intervention seem crucial for optimising clinical outcomes [8]. Recommendations for tailoring interventions emphasise several components, including Comprehensive Geriatric Assessment (CGA) which is a coordinated, multidisciplinary collaboration that assesses the medical, psychosocial and functional capabilities and limitations of an older person [9], family involvement [10], proactive discharge planning [11], and coordinated care with primary care providers [8].

Technological advancements in surgical techniques, an increasing group of older persons, and better comorbidity control have contributed to more surgical treatment among older persons [12]. In persons referred to orthopaedic surgery, pre-admission interventions, such as prehabilitation, have emerged as a proactive strategy to prepare patients for major surgeries to improve outcomes [13]. In older persons with cancer, prehabilitation aiming to optimise patient health and resilience before surgery can mitigate postoperative complications and improve functional recovery [14, 15]. A systematic review of older persons with frailty showed that multimodal prehabilitation, containing training, nutrition support, psychological interventions, or geriatric consultations alone or combined with a preoperative optimisation strategy, positively affected postoperative complications and functional recovery [16]. In older surgical patients, CGA, integrating physical, cognitive, social, and nutrition evaluations, has proven effective in reducing perioperative delirium [17], discharge to an increased level of care [9], and the risk of post-surgery complications [18]. By addressing the multiple dimensions of health and functional status, CGA-based interventions have decreased mortality, improved daily living activities [19], and decreased risk for severe postoperative complications in older surgical patients with frailty [20].

Although CGA has been implemented in various hospital settings [21], we need to gain knowledge of its application in prehabilitation for older patients with frailty before undergoing elective surgery. Currently, there is no described set of principles of prehabilitation utilising CGA alongside training and nutrition support for older persons with frailty undergoing elective orthopaedic surgeries. Therefore, there is a need to develop a multicomponent intervention consisting of evidence-based principles of prehabilitation and perioperative care based on CGA. All components are consistently included, but their content is tailored to the older individual’s needs and circumstances. To establish the effectiveness of this approach, we plan a randomised controlled trial (RCT) to evaluate whether this multicomponent intervention affects the risk of post-surgery complications, enhances interdisciplinary collaboration, and reduces healthcare costs. Prior to the RCT, this mixed-methods feasibility study was conducted to assess the intervention’s feasibility and acceptability aimed at exploring:

1) The extent to which the intended patients were offered the intervention (*reach*),

2) Whether the intervention components were delivered as intended (*dose*),

3) The feasibility of the data collection procedure (*data collection procedure*),

4) How patients and healthcare professionals responded to the intervention (*acceptability*),

5) Whether modifications were made to meet the needs of patients and healthcare professionals (*adaptation*).

## Methods

### Design

The study was conducted from December, 2023, to August, 2024. In this mixed-method feasibility study, we followed the guidelines for reporting non-randomised pilot and feasibility studies [22]. Quantitative and qualitative data were collected simultaneously and analysed separately [23]. The results were synthesised to determine whether and how to proceed with the RCT [24]. Overall, the project was guided by the British Medical Research Council framework for developing and evaluating complex interventions [25].

### Participants and setting

Older persons fulfilling the eligibility criteria were recruited from the Elective Surgery Center, Silkeborg Regional Hospital, Central Denmark Region, a public tax-financed hospital with free access for all citizens when referred by their general practitioner. The inclusion criteria were:

- Age 65 years or above.
- Referred for planned surgery in shoulder, hip, knee, or spine.
- A frailty score of 0.25 or more assessed by the Brief-Multidimensional Prognostic Index (B-MPI) [26].
- Living in Silkeborg Municipality (approx. 100,000 inhabitants).

## The intervention

When developing the intervention, we applied a combined theory and evidence-based approach with patient, public, and stakeholder involvement [27].

*First*, theoretically, we integrated principles from “person-centred care” [7] and “relational coordination” [28]. Principles from person-centred care informed our multidisciplinary emphasis on providing patients with coordinated, personalised, and enabling meaningful and relevant management to their individual lives and needs [7]. Meanwhile, principles from “relational coordination” emphasising shared goals, shared knowledge, and mutual respect among healthcare providers guided our organisational focus on communication and relational processes aimed at task integration [28]. These principles ensured a comprehensive focus on the patient and the corporation between the healthcare professionals.

*Second*, we identified, assessed, and included evidence to support the development of a multicomponent prehabilitation and perioperative care intervention integrating nutrition, exercise, and CGA for older persons with frailty referred to surgery [16].

*Third*, we involved patients and the public by engaging a representative from the local Senior Council in developing and testing the intervention [25, 29]. The representative advocated for the interests of older persons with frailty, contributing to the identification of research topics, intervention components, and outcome selection, as well as providing feedback on participant information and consent forms. During the feasibility study, the representative also advised on intervention adjustments.

*Fourth*, we engaged a geriatric nurse, an orthopaedic physiotherapist, and a surgery pathway coordinator to ensure clinical applicability. Monthly meetings were held with the research team, patient representative, and healthcare professionals throughout the development and implementation phases. A steering committee, comprising hospital department heads, a general practice consultant, and a representative from Silkeborg Municipality, was involved from the outset of the intervention development starting 15 September 2023. The committee met biannually to provide strategic oversight and support implementation.

**Fig. 1:**
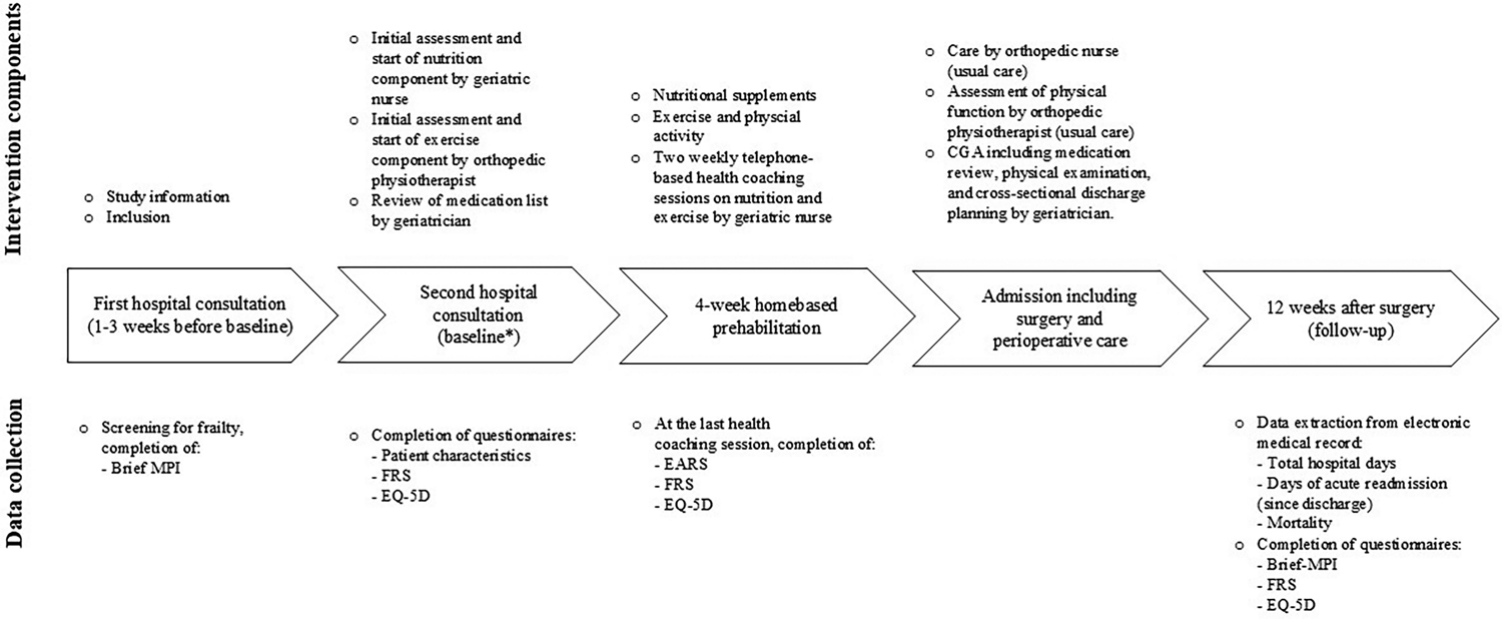
Timeline illustrating the intervention component delivered and the data collection. Abbreviations: Brief MPI: Brief -Multi dimensional Prognostic Index, FRS: Functional Recovery Score, EQ-5D: European Quality of life-5 Dimensions, EARS: Exercise Adherence Rating Scale, CGA: Comprehensive Geriatric Assessment. *Start of the 4-week home based prehabilitataion

In brief, the interdisciplinary multicomponent intervention consisted of a 4-week prehabilitation component aimed at optimising nutrition, promoting exercise, and reviewing the patient’s medication list, and a perioperative care component provided during hospitalisation in collaboration with the patient’s relatives and/or home care services. A detailed description of the template for intervention description and replication (TIDieR) checklist [30] is provided in Table 1, and a timeline illustrating the sequence of the intervention and the outcomes collected is provided in Fig. 1.

**Table 1:** A detailed intervention description according to the template for the intervention description and replication (TIDieR) checklist [30]

1. Brief name	SuperCareGeriatri: multicomponent prehabilitation and perioperative care in older persons with frailty.
2. Why	The intervention was developed on existing evidence on prehabilitation components, comprehensive geriatric assessment (CGA), and perioperative-, geriatric-, and interdisciplinary care. Each component has been shown to impact older persons’ health after surgery positively. However, no randomised controlled trials (RCTs) have assessed the effects of combining the methods in a multicomponent intervention. Therefore, after developing the multicomponent intervention and in line with the Medical Research Council framework, we assessed its feasibility through a mixed-method design prior to conducting the RCT.
3. What - materials	Materials handed to the patients: • Two booklets with individual recommendations highlighting the importance of nutrition and exercise before surgery: one focuses on weight gain, the other on weight maintenance. • Individual exercise programs with exercises illustrated with pictures and brief descriptions.
4. What - procedures	Older patients referred for planned orthopedic surgery were screened for frailty using the Brief Multidimensional Prognostic Index (Brief MPI), which includes areas such as functional ability, nutrition status, medication use, comorbidities, and social factors. Patients with a frailty score of 0.25 or more were offered SuperCareGeriatri which is an interdisciplinary multicomponent intervention consisting of a prehabilitation component and perioperative care component provided during hospitalisation.
	*Prehabilitation* 1. Nutrition: The initial nutrition assessment included focus on protein-rich nutritional supplements and relevant supplements, tailored to the individual patient’s appetite, chewing and swallowing function, dental health, cognitive abilities, and psychosocial conditions. Based on this assessment, the initial component of the intervention, comprising recommendations for nutrition intake, was provided.
	2. Exercise: The initial assessment focused on physical function, activity, and participation based on the International Classification of Functioning, Disability, and Health (ICF). This included a brief functional examination (e.g., gait observation, ability to rise from a chair, and general mobility), supplemented by questions about daily activities such as getting in and out of bed. Based on this assessment in combination with the diagnosis, type of surgery, rehabilitation goals, and motivation, the initial component of the intervention was tailored. Patients received individualised instruction in functional training, such as exercises to improve sit-to-stand performance or correction of gait asymmetry (e.g., encouraging equal weight-bearing when walking). Instruction in postoperative exercises was provided to ensure patient safety and minimize the cognitive load associated with learning new tasks after surgery. Additionally, the identification and ordering of necessary aids were addressed, and the postoperative rehabilitation process was discussed and planned.
	Health coaching with a focus on nutrition and exercise: Two weekly telephone-based health coaching were performed. The coaching included a focus on advice and motivation to maintain a protein-rich nutritional intake, including suggestions for alternative snacks, recommendations for multivitamins, and guidance on avoiding alcohol and tobacco for at least two weeks before surgery. Further, the coaching session included follow-up on exercise goals and motivation to maintain exercise. In addition, other topics such as chronic pain, and low confidence in one’s own abilities were addressed. The content of the coaching session was tailored to each patient’s needs and progress.
	3. CGA including review of medication list: If needed the medication list was adjusted, and recommendations were forwarded to the patient’s general practitioner.
	*Perioperative care* 1. Advice on nutrition, wound care, pain management, urination, and digestion, and focusing on discharge plans in close collaboration with the patient and relatives*. The nutrition advice repeated and reinforced the guidance already given during the prehabilitation phase to ensure consistency and patient adherence.
	2. Assessment of physical function, early mobilization, use of aids, exercise guidance, and possible referral to rehabilitation in the home municipality*.
	3. CGA including medication review and physical examination, and cross-sectional discharge planning. A discharge letter is send as a part of usual practice^*^, as an addition the discharge letter also included results from the CGA. *Identical with usual care
5. Who provided	SuperCareGeriatri was delivered by an interdisciplinary staff in close collaboration with the older person with frailty and their relatives, if any.
	*Prehabilitation* 1. Geriatric nurse from Medical Diagnostic Center, Silkeborg Regional Hospital. 2. Orthopedic physiotherapist from Elective Surgery Center, Silkeborg Regional Hospital. 3. Geriatrician from Medical Diagnostic Center, Silkeborg Regional Hospital.
	*Perioperative care* 1. Orthopedic nurse from Elective Surgery Center, Silkeborg Regional Hospital. 2. Orthopedic physiotherapist from Elective Surgery Center, Silkeborg Regional Hospital. 3. Geriatrician from Medical Diagnostic Center, Silkeborg Regional Hospital.
6. How	*Prehabilitation* The initial assessments of nutrition and physical function, and the first component of the intervention were provided face-to-face. In the 4-week home-based prehabilitation period, health coaching was provided by telephone. Review of medication list was conducted without the patient being present.
	*Perioperative care* Points 1–3 were delivered face-to-face during admission.
7. Where	All components were delivered either at Silkeborg Regional Hospital or by telephone.
8. When and how much	*Prehabilitation* The initial assessment of nutrition and physical function took place four weeks before surgery and lasted approximately one hour. Homebased prehabilitation lasts four weeks and included up to two weekly health coaching sessions (20 min each). The medication review was also carried between initial assessment and surgery.
	*Perioperative care* Points 1–3 were performed on the day of surgery and the first postoperative day, with a total duration of approximately 1.5 h.
9. Tailoring	SuperCareGeriatri was tailored to each patient’s characteristics, disease progression, motivation, and other specific needs. Guidance was provided through active listening and asked questions to support behavioral change e.g. – *“What kinds of snacks with protein do you think you could add to your daily routine?”* (nutrition) – *“What type of exercise do you enjoy and feel confident you can continue with before surgery?”* (exercise).

## Feasibility and acceptability

*Reach* was defined as whether the intended patients were offered the intervention [31] This was examined by describing: (i) screening rate, defined as the percentage of older persons screened for frailty; (ii) recruitment rate, defined as the percentage of older persons with frailty recruited, and (iii) consent rate, defined as the percentage of older persons with frailty who consented to participate in the intervention [32]. 

*The dose* was defined as the quantity of what was implemented [31]: i) the number of conducted phone-based health coaching sessions, ii) the quantity of exercise sessions performed measured by the Exercise Adherence Rating Scale (EARS) (Fig. 1), where high score indicates high adherence [33, 34], and iii) the number of geriatric assessments of the patient’s medication list, and the number of changes to this.

To assess trends over time, the outcomes chosen were total hospital days, days of acute readmission and mortality. In addition, we collected patient-reported outcomes in terms of change in frailty score (B-MPI) [26], physical function measured by the Functional Recovery Score (FRS) [35], health-related quality of life measured by EQ visual analogue scale (EQ VAS) as a part of the EuroQoL 5-Dimension 5-Level (EQ-5D-5 L) [33, 34]. Figure 1 illustrates the measurement timepoints.

We assessed acceptability and adaptation to explore the patients’ and healthcare professionals’ experiences of the intervention, based on data collected through qualitative interviews. We sought to understand the factors influencing their i) perceptions of the intervention’s appropriateness, ii) its alignment with real-world needs, and iii) how it was adapted.

## Data collection procedure

For the quantitative part, all data was entered into and stored in an electronic REDCap database [36]. Data on *reach*,* total hospital days*,* days of acute readmission* and *mortality* was collected from the electronic medical record in the Central Denmark Region. Information regarding *dose*,* patient characteristics* and *patient reported outcomes* were entered into the REDCap database by the geriatric nurse who collected them (Fig. 1).

The qualitative data were collected through interviews with older persons with frailty and healthcare professionals. Five older persons with frailty were pragmatically sampled (three females, mean age 74.8, B-MPI frailty score between 0.31 and 0.44). They accepted participation in individual semi-structured interviews 1–2 weeks post-surgery. ALD conducted these interviews between March and May 2024. Group interviews were conducted with two geriatric nurses and two orthopaedic physiotherapists, while one geriatrician were interviewed individually. CBR conducted the group interviews in August 2024. The healthcare professionals were five females, aged between 27 and 52 years, with several years of experience in their respective geriatrics, physiotherapy, or geriatric medicine fields.

### Data analyses

Descriptive statistics were used in the quantitative analysis. STATA version 17 (Stata, College Station, TX, USA) was used for analysis.

For the qualitative analysis, all interviews were audio recorded and transcribed verbatim. We conducted a descriptive analysis as outlined by Sandelowski 2000 [37], aiming to stay close to the surface of the data and provide a rich, low-inference account of participants’ experiences. ALD and CBR initially familiarised themselves with the data and applied descriptive coding to identify patterns and themes relevant to the intervention’s feasibility, *acceptability*, and *adaptation*. Through iterative discussions between ALD and CBR, the data was organised into core themes reflecting how the intervention was conducted, received and adapted. All authors reviewed and refined the final themes to ensure they accurately represented the data. This descriptive analysis offered detailed insights into how the intervention was conducted, received, and adapted [25]. Interviews ranged from 17 to 47 min, with an average of 34 min.

## Number of participants

Based on organisational variations and considering available resources, we decided to include 14 patients. This was considered suitable for capturing a broad representation of older persons with frailty, generating sufficient data on the feasibility, and accounting for potential dropouts.

## Results

**Fig. 2:**
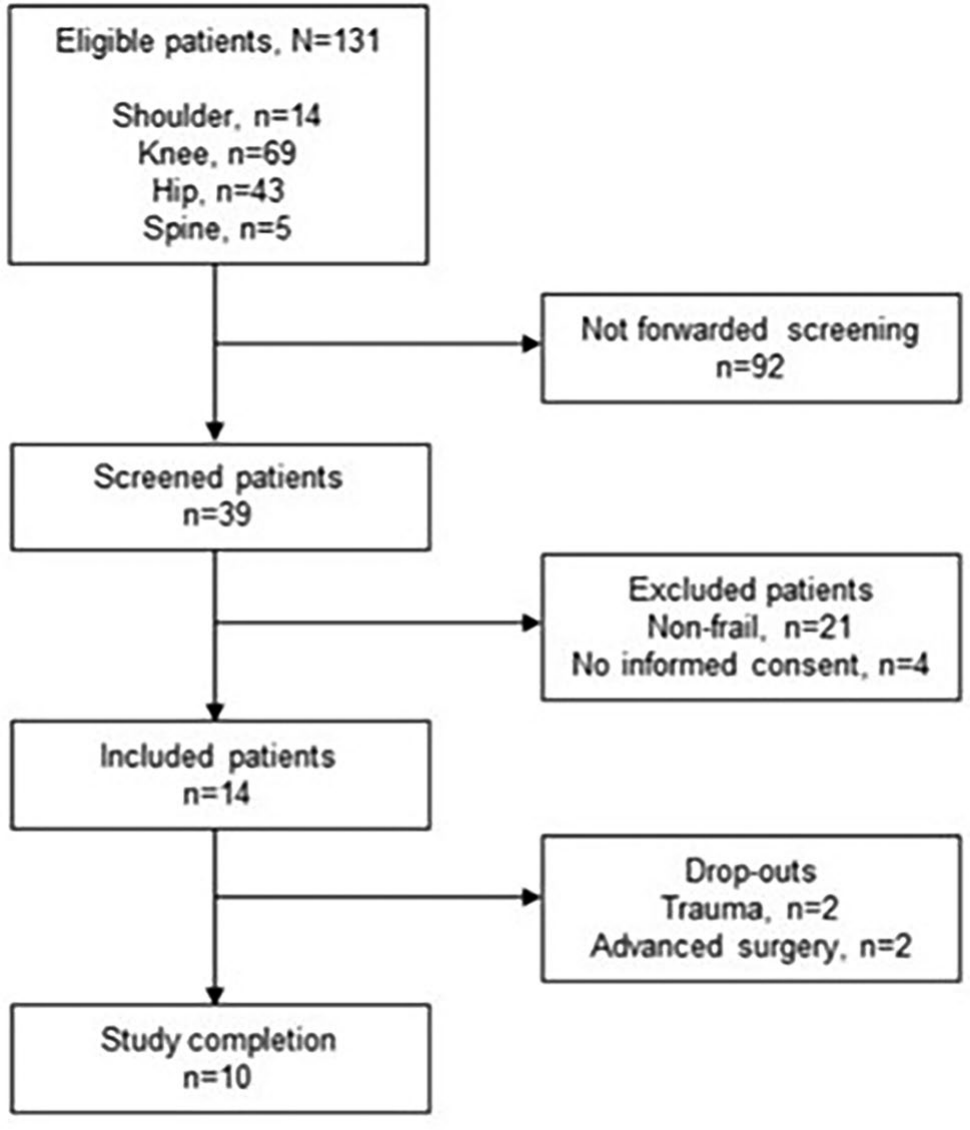
Flowchart of eligible, screened and included patients in the feasibility study.

Considering *reach*, 39 out of 131 (30%) older persons were screened for frailty. Due to an oversight, 92 patients were not forwarded screened. Of the 39 screened at the first hospital consultation, 18 (46%) were frail. Of those, 14 (78%) provided consent to participate (Fig. 2; Table 2). Ten (71%) completed the intervention and follow-up.

According to *dose* throughout the 4-week intervention period, the older persons with frailty received an average of six of the eight planned phone calls (health coaching sessions) from the geriatric nurse. In the interviews all participants reported an increased intake of protein-rich nutritional supplements and consumed primarily milk, protein drinks, curd, and cheese. Exercise adherence was, on average 21 points (SD 4.4) (range: 12–24) indicating an adherence rate of 88%. The 10 patients received a mean of 11.7 drugs (SD 2.3) at the second hospital consultation (Fig. 1). The planned medication review by a geriatrician led to a change in one drug for 5 patients (50%).

**Table 2:** Characteristics of older persons with frailty included in the feasibility study.

	Participants n = 14
Age mean(SD)	76 (9)
Females, n(%)	11 (79)
Living alone, n(%)	10 (71)
Education, n(%)	
$\leq$ 7 years	4 (29)
10 years	4 (29)
High school or further education	4 (29)
Missing	2 (13)
Locatrion of surgery, n(%)	
Shoulder	0 (0)
Knee	7 (50)
Hip	4 (29)
Spine	3 (21)
Number of drugs, n(%)	
0–3	0 (0)
4–6	3 (21)
7+	11 (79)
Body Mass Index, n(%)	
< 21 or $\geq$30	8 (57)
Physical function (FRS), mean (SD)	89 (13)
Degree of frailty (B-MPI), n(%)	
Pre-frail	4 (29)
Moderately frail	10 (71)
Severely frail	0 (0)

SD: Standard Deviation

FRS: Functional Recovery Score, range 0–100 (100 = best)

B-MPI: Brief Multidimensional Prognostic Index (pre-frail: 0.25–0.33, moderately frail: 0.34–0.066, severely frail: 0.67–1.00)

The *data collection procedure* was successful, as all outcomes from the electronic medical records and the patient-reported outcomes were obtained on all 10 completing patients. At 12-week follow-up, the median length of hospital days was 1 day (interquartile interval 1–3 days). One participant was acutely readmitted twice due to surgical wound infection and constipation, respectively. Physical function improved by three points on the FRS scale (0-100 scale), and quality of life improved by 9.5 points (SD 14) on the 100-point EQ-VAS. Frailty status remained unchanged. No harm or unintended consequences were identified during the feasibility study (Table 3).

The qualitative analysis identified four themes that described the *acceptability* and *adaption* of the intervention: Individualised intervention, Support systems, Collaboration and synergy, and Adaptation during the study.

**Table 3:** Trends over time in outcomes following intervention in older persons with frailty

	Participants n = 10
Total hospital days*, median (iqr)	1 (1–3)
Readmission**, n(%)	1 (10)
Change in physical function***, mean (sd)	3 (7.3)
Change in frailty status ****, mean (sd)	− 0.05 (0.21)
Change in quality of life***, mean (sd)	9.5 (14)
Deaths *, n(%)	0 (0)

*From admission to 12 weeks after surgery

**From discharge to 12 weeks after surgery

***From second hospital consultation (baseline) to 12-weeks after surgery

****From first hospital consultation to 12 -week after surgery

Iqr: interquartile range

Sd: Standard deviation

Physical function measured by Functional Recovery Score, range 0–100 (100 = best)

Frailty status measured by Brief-Multidimensional Prognostic Index,range 0–1 (1 = best)

Quality of life measured by EQ-5D, 0-100 (100 = best)

### Individualised intervention

A significant theme across interviews with the older persons with frailty and the healthcare professionals was the possibility of tailoring the prehabilitation to the older persons with frailty needs and preferences. Healthcare professionals emphasised that the structured approach made building strong relationships with patients possible, allowing for a personalised, in-depth understanding of their circumstances. As one orthopaedic physiotherapist explained, “*I really appreciate taking the time to get to know the patient before intervening*,* especially when we’re dealing with older*,* vulnerable individuals and exploring the challenges they might face […] In the initial consultation*,* they’re often inclined to hold back on certain things or put a positive spin on them*,* but when we have them there [in the clinic] and test certain things and talk to them about what they actually eat*,* what they really weigh*,* and what they genuinely do throughout the day*,* the truth comes out*,* and we gain a better understanding of them*” (Orthopaedic physiotherapist 1).

A key point was that patients often downplayed their constraints. However, through individualised assessments, the healthcare professionals were able to uncover significant barriers such as poor nutrition, limited physical function, or the strain of caregiving responsibilities, especially for those with ailing relatives. Understanding patients’ situations enabled more meaningful and individualised dialogue, including introducing practical aids like rollators and discussing postoperative support at home. Although the geriatricians primarily engaged with patients post-operatively, the intervention’s framework enabled them to better tailor care to each individual patient.

Regarding nutrition, the older persons with frailty appreciated the practicality of the nutritional coaching, which helped them incorporate protein-rich food into their daily routines. One participant shared, *“I got a list of protein-rich products*,* and they were all available at the grocery store”* (P13). Healthcare professionals recognised the importance of making nutritional recommendations easy to follow, which increased patient adherence. Some patients struggled to meet nutritional goals, prompting the geriatric nurses to adjust the plans to better fit individual lifestyles and preferences. For example, one patient who could not manage three meals daily was encouraged to increase her milk intake to boost protein. The geriatric nurses experienced the positive effect of making the intervention less overwhelming, emphasising small, manageable nutritional changes. The coaching appeared to have a lasting impact as participants continued to apply the knowledge and behaviour changes post-intervention. For example, one older man reported still following the nutritional advice, saying, “*I drink more milk than I otherwise would have done*,* and I’ve eaten those yoghurt-like products. Whether it helps*,* I don’t know*,* but I have no problems with my wound or healing”* (P13).

Participants had varying preferences for exercise - some favoured exercise in social settings and others in individual sessions. Tailoring the exercise program facilitated higher engagement and feasibility. Finding motivation was a challenge and barrier for participants who had not previously engaged in exercise. Some participants desired more opportunities to meet with the orthopaedic physiotherapist during the program. For instance, one patient mentioned, “*It would have been nice to come in occasionally and see the physiotherapist to check if what you’re doing is correct*” (P15).

The orthopaedic physiotherapists also stressed that allowing patients to set their goals and choose how to participate in the program significantly improved motivation and adherence. As one orthopaedic physiotherapist explained, “*We can easily give them a bunch of exercises that they won’t do*,* just because we think it could be good for them*,* but they also need to see the point of doing these things*” (Orthopaedic physiotherapist 2). The orthopaedic physiotherapists highlighted that the intervention allowed them to assess and interact with patients before surgery, which was invaluable for understanding their specific challenges and needs. This early interaction allowed them to identify issues that would otherwise have gone unnoticed, helping them tailor advice and exercise plans to everyone’s capabilities and barriers.

### Support systems

Support from healthcare professionals and relatives played a crucial role in patient adherence. Many participants relied on weekly check-ins from the geriatric nurses to stay on track. One patient reflected, “*It was really good that Eva called once a week to ask how I’m doing and if I’m taking the protein I should*” (P8). These regular interactions provided reassurance and a sense of accountability, motivating patients to follow the program. However, geriatric nurses observed that patient needs varied regarding the frequency of follow-ups. Some found the twice-weekly calls excessive, leading healthcare professionals to adjust their approach, particularly during the later weeks of the intervention.

Although bringing a relative was encouraged in the standard invitation letter for consultations in the orthopaedic surgery department, the geriatric nurse also explicitly encouraged patients to bring a relative during the first consultation in the intervention, which was scheduled after the initial surgical consultation. This additional encouragement aimed to ensure that patients had support from family members, but not all patients were accompanied. Healthcare professionals noted that those with support from relatives experienced a smoother recovery process and suggested more explicit encouragement for patients to involve family members, as this support was essential in managing the intervention’s demands. One patient commented, “*It would have been difficult without my daughter’s help*” (P8).

Healthcare professionals highlighted that a lack of communication with local municipalities hindered the ability to plan for patient care needs post-discharge, which was especially problematic for older persons without relatives. Further, some patients were overly dependent on relatives, e.g., when a non-Danish-speaking patient preferred using a daughter as an interpreter rather than the services offered. A small co-payment requirement partly influenced the patient’s preference. However, this reliance complicated the process, as the relative was only sometimes available at the scheduled times. This situation underscored the importance of consistent and accessible interpretation services to ensure regular and effective communication.

### Collaboration and synergy

The intervention fostered stronger interdisciplinary collaboration among healthcare professionals. The interviewed healthcare professionals emphasised that the interdisciplinary approach created a more cohesive and supportive patient experience. They learned from each other’s expertise, which improved their ability to provide a more integrated approach to care. One orthopaedic physiotherapist highlighted, “*The collaboration meant we could adjust care plans in a more integrated way*” (Orthopaedic physiotherapist 2).

This interdisciplinary approach allowed for a more comprehensive understanding of patient needs, aligning with the intervention’s goals of providing person-centred care.

### Adaptations to procedures

As the study progressed, healthcare professionals identified areas for potential intervention improvements. One adaptation suggested was for the geriatric physician to meet with patients before surgery to assess their needs. This approach aimed to address limitations earlier. As the geriatric physician proposed, “*If I could wish for something*,* it would be to have an outpatient clinic set up so that I could be linked to it as a sort of supervisor*,* who could just be pulled in to look at certain aspects while the patient is there*,* because that would really improve the quality*” (Geriatrician).

It took time for healthcare professionals to fully integrate recruitment into their workflow. Thus, we began to visit the pathway coordinator at the orthopaedic ward each morning, and as the study progressed, recruitment rates improved. One geriatric nurse reflected, “*At first*,* we did not always remember to recruit patients*,* but by the end*,* it became a natural part of our routine*” (Geriatric Nurse 2). Additionally, baseline data were initially collected face-to-face. Questionnaires were conducted by phone the day before surgery and again 12 weeks after surgery. However, all questionnaire data were collected face-to-face for the non-Danish-speaking participants to ensure clarity and understanding.

Although the primary aim of the phone calls was nutrition and exercise coaching, the intervention expanded to include practical guidance on surgery preparation. Patients also raised broader questions, prompting geriatric nurses to investigate or refer them to other services. This shift in role reflects the need for both the physical and logistical aspects of surgery preparation.

## Discussion

### Summary of main findings

We feasibility tested a multicomponent 4-week home-based prehabilitation program and perioperative care intervention for older persons with frailty referred to planned orthopaedic surgery. The screening rate was as low as 30%. The recruitment rate was 46%, and the consent rate 78%. In total, 71% completed the intervention and follow-up. The number of telephone consultations was reduced from eight to six on average. In addition, we found that 100% of the participants followed the advice to consume protein-rich dairy products, 88% adhered to home-based exercise and each medication review led to medication adjustments. Several *adaptations* were implemented immediately, while others were proposed to inform the design of a future RCT. Further, the intervention was found *acceptable* by both the older persons with frailty and the healthcare professionals.

### Comparison with existing literature

 Feasibility studies on prehabilitation in frail older orthopaedic patients have previously been conducted. A single-blind pilot RCT from the Netherlands examined the feasibility and preliminary effectiveness of a 3–6-week home-based intensive exercise program for frail older patients awaiting elective total hip arthroplasty [38]. Thirty frail older patients were included. Patient satisfaction and adherence to the program were high, and no serious adverse events were reported. Moreover, participants in the intervention group showed a statistically significant improvement in the 6-minute walk test compared to the control group [38]. Similar to our study, the training was performed at home prior to surgery, with high adherence rates. Notably, the Dutch study showed that older adults were willing to participate and even expressed satisfaction with the intervention. Before initiating a larger experimental trial, it is essential to establish that frail patients are not exposed to undue risks, such as falls. In our study, two patients dropped out due to traumatic falls that occurred shortly after their inclusion. To strengthen preventive efforts, we suggest introducing the Falls Efficacy Scale–International (FES-I) to assess fear of falling in future interventions including frail older persons [39].

Similarly, a recent feasibility study in the UK investigated frail patients undergoing hip and knee replacement [40]. The participants were encouraged to perform daily home exercises for 12 weeks prior to surgery, provided with a daily protein supplement, and supported through six telephone calls during the intervention period. Compared with the UK study, which lasted three times longer and included a cohort six times larger, we achieved higher adherence rates for exercise (88% vs. 68%). Our qualitative findings indicated that the nutritional recommendations were well-received compared to a 58% adherence rate in the UK study. This suggests that our shorter intervention was more accessible. Despite differences in duration and size, both studies demonstrated that such interventions are feasible for frail older patients prior to surgery.

In older cancer patients, experimental studies have been conducted. An RCT of preoperative geriatric assessment followed by a tailored multimodal 3-week intervention included nutrition, exercise and medication review in patients scheduled for elective colorectal cancer surgery [41]. The intervention components were comparable with ours. In terms of recruitment rate, they assessed 264 patients, of which 160 (61%) were frail, which is slightly higher than our recruitment rate (46%). Regarding consent rate, they found that 122 (91%) provided consent, whereas we obtained a lower consent rate, reaching 78%. In the intervention group, 53 (93%) completed a 30-day follow-up compared to 10 (71%) patients completing our study. A similar study, the PREHAB trial [42], evaluated a home-based prehabilitation program that found no improvement in postoperative recovery or other outcomes among older adults with frailty undergoing cancer surgery. The mean adherence rate in that study was 61%, which was lower than in our study. This difference in adherence may be a key mediator of prehabilitation efficacy.

A non-randomised cohort study in older persons undergoing elective colectomies due to cancer introduced an intervention consisting of three weeks of nutritional supplementation and resistance exercise with weekly review by the physiotherapies, and one geriatrician consultation to optimise for polypharmacy and other geriatric aspects [43]. There was no indication of screening for frailty. However, the intervention is identical to the one we tested for feasibility. They were able to recruit 58 suitable patients (denominator unknown), and they all completed the intervention. This is more successful than the 14 (78%) who provided consent and 10 (71%) who completed our study.

A study found that 95% of older persons with frailty undergoing elective inpatient major non-cardiac, non-neurologic or non-orthopaedic surgery were willing to participate in a home-based, multimodal prehabilitation programme, and that 87% of participants considered the exercise program easy to follow, enjoyable, and well-suited to their needs [44]. Thus, their consent rate was slightly higher than our 78%, but their findings in terms of acceptability were similar to ours.

We are aware that similar studies to ours are ongoing. One protocol describes an upcoming study where the study population differs from ours, but the intervention is nearly identical [45, 46] whereas another study includes a similar study population but has a longer duration of intervention [40].

### Discussion of findings, including adaptations

No formal cut-off criteria exist for reach; however, with a screening rate of 30%, efforts should focus on enhancing this rate before and during the RCT. The high proportion of frailty among those screened suggests that screening was conducted consecutively, but rather selectively. When we became aware of the low screening rate, an *adaptation* was made as we began to meet with the pathway coordinator at the orthopaedic ward each morning. This integrated the screening for frailty into healthcare professionals’ daily workflow, thereby improving the screening rate at the end of the study period. This phenomenon has been described before [47]. Nevertheless, the initially low reach highlights a challenge that may have affected the validity of the findings, as those not screened or invited may have had different characteristics than those included. Another issue was that four of 14 patients did not complete the intervention despite agreeing to participate. Two had a traumatic fall during the intervention period. Therefore, we introduced the screening tool Falls Efficacy Scale-International to measure the fear of falling [39]. This made it possible for us to initiate preventive initiatives, e.g., recommending a rollator.

A wide range of barriers and facilitators may affect patients’ ability to participate in prehabilitation. Addressing these factors requires careful considerations of each patients’ capability, opportunity and motivation [48]. In our study, regular health coaching by geriatric nurses were essential in maintaining participant motivation and accountability and providing support and reassurance.

In terms of *dose*, the planned number of phone calls was reduced from eight to an average of six, based on feedback from older persons with frailty who found weekly health coaching to be excessive, particularly in the later stages of the intervention. This *adaptation* improved *acceptability* by making it more manageable for participants.

Future *adaptation* could further personalise the frequency and timing of health coaching to better align with individual needs, for example, by designing follow-up schedules that balance patient preferences and clinical resources, thereby promoting realistic and effective engagement and enhancing the overall *acceptability*.

We were able to collect data on all ten completed patients, making the *data collection procedure* successful. The total follow-up was partly due to an *adaptation* concerning the *data collection procedure* for the patient reported outcomes. Instead of completing questionnaires via email as planned, they were completed by phone or face-to-face with the geriatric nurse before and after surgery; however, for a non-Danish-speaking participant, all data were collected face-to-face. This approach ensured complete response rates.

Overall, participants reported high engagement, largely due to the intervention’s individualised approach, which allowed them to incorporate nutrition and exercise guidance into their routines. The qualitative finding indicated that the nutrition component was well-received, as protein-rich foods could be integrated with minimal disruption to daily life, enhancing adherence even beyond the intervention period.

Although healthcare professionals observed low motivation among some participants, particularly those new to exercise, this was not reflected in the patient-reported EARS scores, which generally indicated high adherence. Healthcare professionals addressed motivational challenges by involving participants in personalised goal setting, including exercises aligned with their priorities and capacities. Involving patients in setting their own goals has been linked to improved functional outcomes and greater goal attainment in rehabilitation [49] similar benefits may be expected in prehabilitation.

This approach appeared to enhance motivation and adherence, as participants were more inclined to engage in activities that were meaningful to them. Emphasising patient involvement reinforced the idea that empowering individuals to shape their own health behaviours increase the acceptability of prehabilitation. Both patients and healthcare professionals valued the tailored components such as exercise programmes and nutritional guidance, which supported engagement by aligning the intervention with each participant’s needs and circumstances.

A key strength of the intervention was its emphasis on interdisciplinary collaboration, which facilitated the development of comprehensive care plans that more effectively anticipated and managed patients’ needs pre- and post-operatively. The observed integration suggests that a more formalised inclusion of a multidisciplinary team (MDT), including the orthopaedic surgeon, could further strengthen the intervention [28]. In addition, expanding collaboration across healthcare sectors could enhance continuity and relevance. For instance, involving general practitioners in frailty screening at the time of referral may improve the identification of suitable candidates. Given that much of the prehabilitation occurs in patients’ homes, it may also be relevant to involve municipal physiotherapists in supervising daily exercise routines in collaboration with hospital-based staff.

Prehabilitation is a resource-intensive yet promising strategy for supporting frail surgical patients and requires organizational readiness and adaptations to local context [50]. An RCT involving frail older cancer patients highlight that many patients rely heavily on their social network, particularly for transportation and emotional support and that a lack of such support could compromise adherence and perceived benefit [51]. Our findings echo this, as support from relatives was a key factor in maintaining engagement and following the programme.

This underlines the importance of developing alternative support structures for patients without family assistance. Healthcare professionals in our study highlighted the potential value of community-based services to promote equitable access. Furthermore, challenges related to language barriers were evident, particularly for non-Danish-speaking patients who relied on family members as interpreters. This reliance often caused logistical difficulties, suggesting a need for professional interpretation services to improve communication and engagement across the patient population. Addressing these social and language-related factors will be essential to ensuring the intervention is both accessible and inclusive.

Four requests for *adaptations* to the intervention and study procedures in the future RCT were identified: (1) healthcare professionals proposed that geriatricians should be included earlier in the process, as part of the MDT during the initial CGA, to assess medication, fall risk, and individual needs, potentially preventing complications during prehabilitation. The remaining three requested adaptations were raised by the research team, (2) limiting inclusion to patients referred for planned knee and hip surgery, as these pathways are organisationally identical, allowing for better standardisation, (3) a follow-up phone call from the geriatric nurse 48 h after discharge was recommended to address early post-discharge concerns, provide reassurance, and support continuity of care, and (4) initiating dialogue with general practitioners to support frailty screening prior to surgical referral, and improved integration with municipal rehabilitation services. All four requests will be considered before to the RCT.

### Strengths and limitations

This study employed a mixed-methods feasibility design, allowing for a broad evaluation. One strength was the thorough development of the intervention, grounded in existing evidence and the active involvement of patients and healthcare professionals. This approach ensured that the intervention was based on empiric knowledge and aligned with the practical needs of its users, enhancing relevance and *acceptability*. Further, using data from electronic medical records for most outcomes allowed for complete follow-up.

The mixed-methods approach enabled data triangulation, providing a nuanced understanding of the intervention’s *reach*, *dose*, *data collection procedure*,* acceptability*, and *adaptation*. Quantitative measures offered clear indicators of *reach* and *dose*, while qualitative interviews provided in-depth insights into patient and healthcare professional experiences, shedding light on contextual factors and individual responses that might otherwise have been overlooked.

However, a key limitation was the initially low screening rate, as several eligible patients were not invited due to procedural oversights in the department. Although this was corrected by daily check-ins from a geriatric nurse, which improved recruitment over time, the initial limitation in reach may have influenced the validity of the findings.

Further, the absence of predefined progression criteria can be considered a limitation. However, this decision was deliberate due to several factors: insufficient evidence to establish valid thresholds; a focus on identifying unforeseen challenges and refining the intervention and procedures; the need for flexibility during the feasibility phase; and uncertainty regarding recruitment of frail older adults, which could render percentage-based criteria misleading in a small sample. Instead, feasibility was assessed descriptively to explore strengths, challenges, and areas for improvement.

We need more data on whether older persons with frailty would be willing to participate in a randomised study. Additionally, perspectives from relatives, who played a significant role, were not included. Despite these limitations, this study provides a foundation for refinement before proceeding to a full-scale RCT, with insights into areas requiring adaptation and additional support mechanisms.

### Generalisability

In terms of generalisability, no participants with severe frailty were included, likely due to clinical decisions by general practitioners and/or surgeons not to offer elective surgeries to this group. Consequently, the study population consisted of older persons with pre-frailty and moderate frailty, meaning that the findings may not fit those with severe frailty. Furthermore, the initially low screening rate reduced the study’s reach, which may limit the extent to which the findings can be generalised to all older adults referred for elective surgery.

## Conclusion

This mixed-methods feasibility study demonstrated that the developed multicomponent intervention, based on theory, evidence and patient and public involvement, was feasible and acceptable to both patients and healthcare professionals in the studied setting with the added changes to interventions. Individualised support and interdisciplinary collaboration contributed to patient engagement and adherence. However, the limited reach underline important challenges, necessitating adaptation. Most components were delivered as intended, suggesting that the delivery of the intervention was achievable within this clinical context. Several adaptations were implemented immediately, while others were proposed to inform the design of a future RCT. Findings should be interpreted within the specific clinical setting, and no conclusions can be drawn beyond this context.

## Data Availability

The data supporting this study’s findings are available from the corresponding author upon reasonable request.
